# Activation of the Melanocortin-4 receptor signaling by α-MSH stimulates nerve-dependent mouse digit regeneration

**DOI:** 10.1186/s13619-021-00081-9

**Published:** 2021-05-03

**Authors:** Hanqian Xu, Hailin Zhang, Yanqing Fang, Huiran Yang, Ying Chen, Chao Zhang, Gufa Lin

**Affiliations:** 1 Key Laboratory of Spine and Spinal Cord Injury Repair and Regeneration of Ministry of Education, Orthopaedic Department of Tongji Hospital, School of Life Sciences and Technology, Tongji University, Shanghai, China; 2 Stem Cell Institute, University of Minnesota, Minneapolis, MN 55455 USA; 3 Translational Medical Center for Stem Cell Therapy and Institute for Regenerative Medicine, Shanghai East Hospital, Shanghai Key Laboratory of Signaling and Disease Research, Frontier Science Center for Stem Cell Research, School of Life Sciences and Technology, Tongji University, Shanghai, China

**Keywords:** MC4R, Mc4r-gfp, α-MSH, Digit regeneration, Distal, Proximal, Denervation, Neurotrophic function

## Abstract

**Background:**

Expression of Mc4r in peripheral organs indicates it has broader roles in organ homeostasis and regeneration. However, the expression and function of Mc4r in the mouse limb and digit has not been fully investigated. Our previous work showed that *Mc4r−/−* mice fail to regenerate the digit, but whether activation of MC4R signaling could rescue digit regeneration, or stimulate proximal digit regeneration is not clear.

**Results:**

We analyzed the expression dynamics of Mc4r in the embryonic and postnatal mouse limb and digit using the *Mc4r-gfp* mice. We found that Mc4r-GFP is mainly expressed in the limb nerves, and in the limb muscles that are undergoing secondary myogenesis. Expression of Mc4r-GFP in the adult mouse digit is restricted to the nail matrix. We also examined the effect of α-MSH on mouse digit regeneration. We found that administration of α-MSH in the *Mc4r+/−* mice rescue the delayed regeneration of distal digit tip. α-MSH could rescue distal digit regeneration in denervated hindlimbs. In addition, α-MSH could stimulate regeneration of the proximally amputated digit, which is non-regenerative.

**Conclusions:**

Mc4r expression in the mouse limb and digit is closely related to nerve tissues, and α-MSH/MC4R signaling has a neurotrophic role in mouse digit tip regeneration.

**Supplementary Information:**

The online version contains supplementary material available at 10.1186/s13619-021-00081-9.

## Background

The melanocortin 4 receptor (MC4R) plays a pivotal role in energy balance (Gautron et al., 2015; Krashes et al., 2016; Sternson and Eiselt, 2016). Loss-of-function *MC4R* variants are among the most common genetic causes of early-onset obesity in humans (Vaisse et al., 1998; Yeo et al., 1998), while gain-of-function *MC4R* variants are associated with protection from obesity and its complications (Lotta et al., 2019). In the mouse, targeted deletion of *Mc4r* causes weight gain, early onset obesity and increased linear growth (Huszar et al., 1997). Studies in other animal models, including fish and amphibians, show that MC4R has conserved roles in body weight control (Li et al., 2021; Zhang et al., 2012). Consistently, MC4R level is high in the central nervous system. It is expressed in the paraventricular nucleus of the hypothalamus (PVN), the dorsal motor nucleus of the vagus (DMV), and the intermediolateral nucleus of the spinal cord (IML) (Gantz et al., 1993; Mountjoy et al., 1994; Mountjoy and Wild, 1998; Siljee et al., 2013).

Recent work has shown that MC4R is also involved in other biological processes, such as neuropathic pain control, stress regulation, and cardiovascular function homeostasis (Litt et al., 2017; Zhao et al., 2019). Mc4r is expressed in the enteroendocrine L cells and its activation by α-MSH (α-melanocyte stimulating hormone) leads to the release of PYY (Peptide YY) and GLP-1 (Glucagon-like Protein 1), which then regulates nutrients adsorption (Panaro et al., 2014). Mc4r is also expressed in the rat hepatocyte and its expression level is elevated after partial hepatectomy (Xu et al., 2016). These studies indicate that MC4R has broader expression and functions in the peripheral organ and tissues, outside the central nervous system. But the role of MC4R in those non-neural tissues and organs has not been adequately investigated.

In the limb, Mountjoy et al. reported that *Mc4r* transcripts are present in E12-E18 embryos (Mountjoy et al., 2003). Our work also showed that MC4R signaling is active in the tadpole limb and regulates limb regeneration, and digit tip regeneration is defective in *Mc4r* mutant mice (Zhang et al., 2018). But the cell types expressing Mc4r, particularly in the adult limb of the mouse, have not been thoroughly characterized. While *Mc4r−/−* mice failed to regenerate the digit, *Mc4r+/−* mice could still regenerate the distal digit tip, but with significantly delayed formation of blastema (Zhang et al., 2018). Whether activation of MC4R signaling in the mice could rescue digit tip regeneration has not been explored.

MC4R has high affinity to its endogenous agonists α- and β- MSH (Yeo and Heisler, 2012). Binding of MSH to MC4R activates the MC4R signaling and raises intracellular cAMP levels (Anderson et al., 2016). Thus, in this work, we used α-MSH to activate the MC4R signaling and examined whether it can rescue digit regeneration in the *Mc4r* heterozygous mice. To explore whether α-MSH can function as neurotrophic factor, we denervated the hindlimb and examined digit regeneration after α-MSH injection. We also checked whether α-MSH activation of MC4R can stimulate proximal digit regeneration. We report that Mc4r is dynamically expressed in the mouse limb and digits, and confirm that α-MSH/MC4R signaling has a neurotrophic role in mouse digit regeneration.

## Results

### Mc4r-GFP expression dynamics during mouse embryonic limb development

**Fig. 1:**
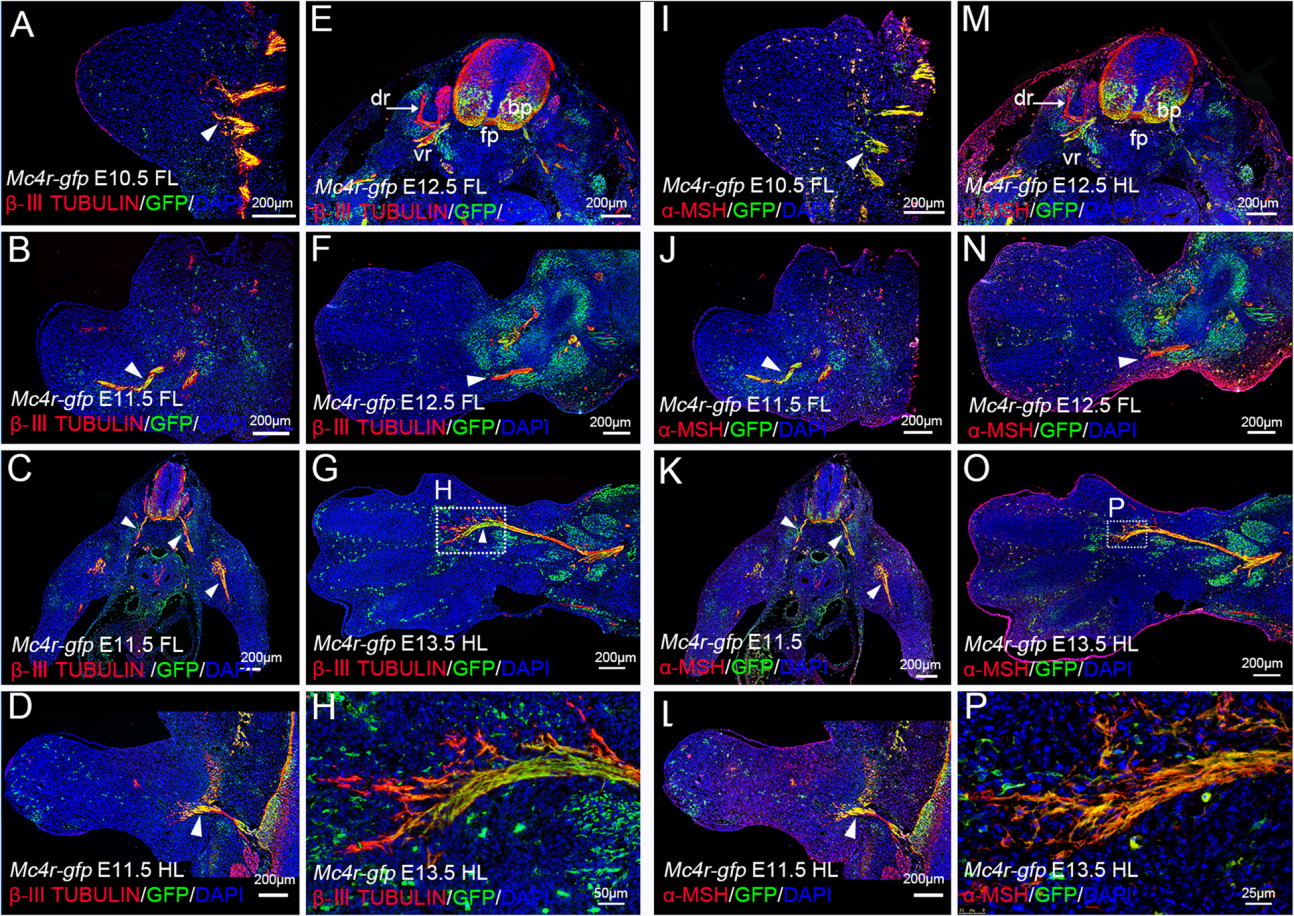
Mc4r-GFP and α-MSH expression in limb nerve tissues. **a-h** Representative images of Mc4r-GFP and βIII-tubulin expression detected by immunofluorescence staining, on sections of E10.5 forelimb (FL) (**a**), E11.5 forelimb (**b,c**), E11.5 hindlimb (HL) (**d**), E12.5 forelimb (**e,f**) and E13.5 hindlimb (**g,h**). **i-p** Images of Mc4r-GFP and α-MSH staining, on sections adjacent to those shown in **a-h**. Sagittal sections of the limb are shown distal to the left, except **c,e,k,m** are transverse sections of the embryo through the forelimb. (**h**,**p**) are high magnification images of areas outlined in (**g**, **o**). Arrowheads indicate Mc4r-GFP positive nerve tissues. Arrows (in **e**, **m**) indicate dorsal ramus (dr). bp, basal plate; vr, ventral ramus; fp, floor plate; FL, forelimb; HL, hindlimb. Scale bars are 200 μm, except for (**h**), 50 μm and (**p**), 25 μm

Previously, we showed that Mc4r, and its agonist α-MSH, is expressed in the nerve tissues of the tadpole limbs, and is required for tadpole limb regeneration (Zhang et al., 2018). To examine the dynamics of Mc4r expression during mouse limb development, we used the *Mc4r-gfp* transgenic mice, which faithfully reports the expression of Mc4r (Liu et al., 2003). Since Mc4r has been shown to be predominantly expressed in the nervous system (Gantz et al., 1993; Mountjoy et al., 1994; Mountjoy and Wild, 1998; Siljee et al., 2013), we started with analysis of Mcr4-GFP in the limb by using βIII-tubulin as a marker for nerves. The mouse limbs begin as bumps on the flank of the E9.5 embryos, with the development of the forelimb precedes that of the hindlimb by about a half day (Martin, 1990). We analyzed both the forelimb and the hindlimb starting at E9.5, and found similar patterns of Mc4r-GFP expression. We did not observe any GFP signals in the E9.5 limb field (not shown) or the E10.5 limb bud, though the spinal nerves are clearly GFP positive at these stages (Fig. 1a). At E11.5, in both forelimb and hindlimb, the spinal nerves about to enter the limb bud are Mc4r-GFP/βIII-tubulin positive (Fig. 1b-d, arrowheads). Expression of Mc4r-GFP in the spinal nerves are more obvious in E12.5 embryos. We observed high levels of Mc4r-GFP expression in the basal plate (bp), the roof plate (rp) of the spinal cord, and the ventral ramus (vr) of the spinal nerves (Fig. 1e,f). But the dorsal ramus is void of Mc4r-GFP expression (arrow in Fig. 1e). By E13.5, the limb nerves have extended intensively into the limb, and established contacts with the muscle masses (Hurren et al., 2015). From this stage on, we observed high level of Mc4r-GFP expression in the limb nerves, including the branches at the leading edges (Fig. 1g,h).

We also analyzed the expression of α-MSH, and observed co-expression of Mc4r-GFP and α-MSH in the nerve tissues (Fig. 1i-p). There is a good correlation of Mc4r-GFP and α-MSH expression in the limb nerves (βIII-tubulin+), indicating that the limb nerves express both α-MSH and MC4R (Fig. 1h, p). Notably, α-MSH is more broadly expressed, with low level of expression in the inter-digit mesenchyme and strong expression in the epithelium. Nevertheless, the high level of α-MSH and MC4R in the limb nerve is consistent to our previous analysis of α-MSH expression in the tadpole limb, which suggests that the α-MSH/MC4R signaling has a neurotrophic role (Zhang et al., 2018).

**Fig. 2:**
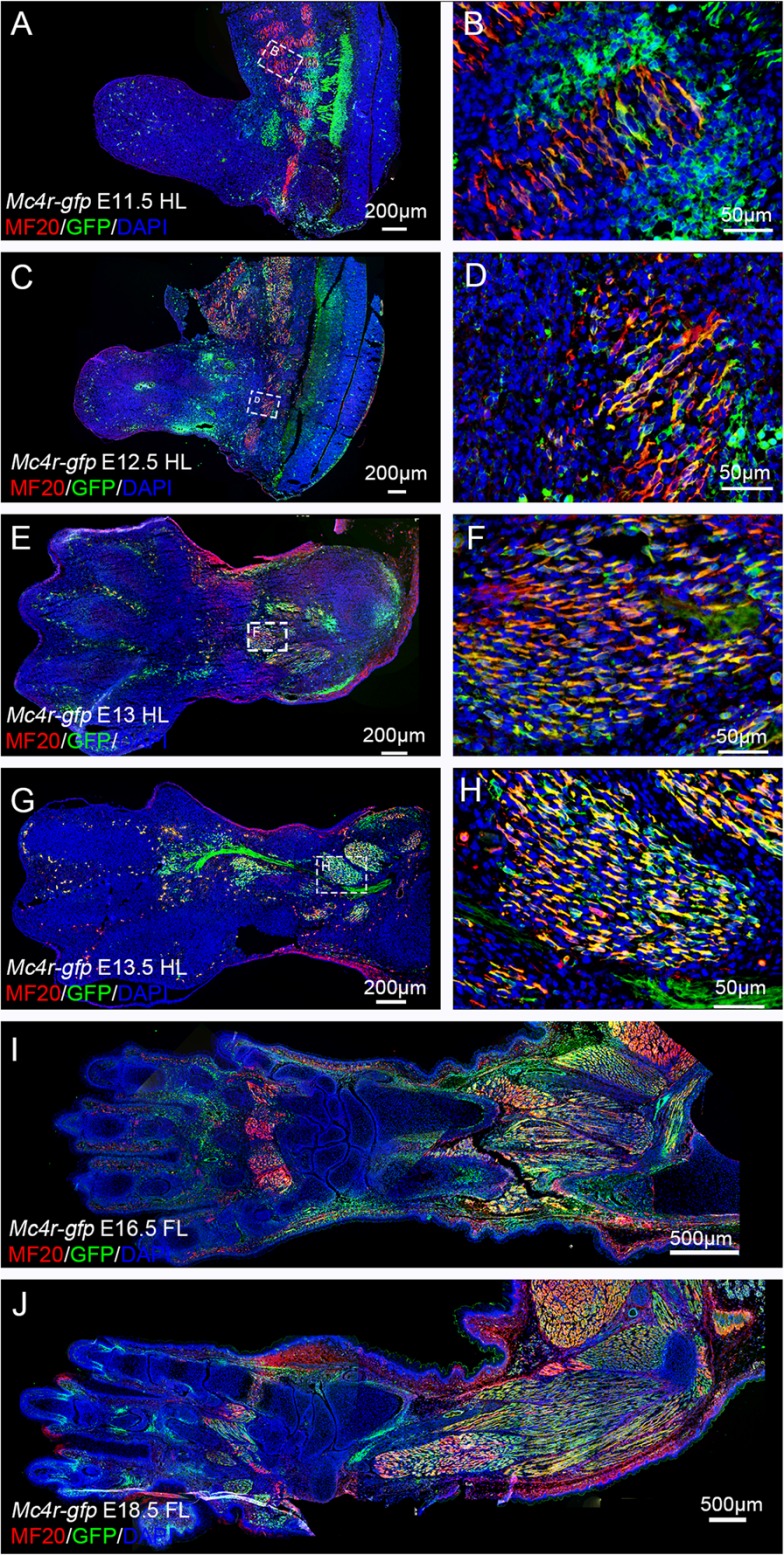
Mc4r-GFP expression in limb muscles. **a-j** Representative images of Mc4r-GFP and MF20 expression detected by immunofluorescence staining, on sections of E11.5 hindlimb (**a,b**), E12.5 hindlimb (**c,d**), E13 hindlimb (**e,f**), E13.5 hindlimb (**g,h**), E16.5 forelimb (**i**) and E18.5 forelimb (**j**). All are sagittal sections of the limb, distal to the left. (**b,d,f,h**) are higher magnification of outlined areas in (**a,c,e,g**). Scale bars: 200 μm in **a,c,e,g**; 50 μm in (**b,d,f,h)**; 500 μm in (**i**, **j)**

In addition to the high level of Mc4r-GFP and α-MSH expression in the innervating limb nerves, Mc4r-GFP expression is also obvious in the proximal muscle mass, from E12.5 onward (Fig. 1f,g). To gain better understanding of Mc4r expression in the limb muscles, we stained the limb sections with MF20 as a marker for muscle cells (Martin, 1990). At E11.5, although the limb bud does not contain any MF20 positive cells, some MF20 and Mc4r-GFP double positive cells are present in the somites (Fig. 2a,b). This is more evident at E12.5 (Fig. 2c,d). At E13.5, both the forelimb and hindlimb have accumulated large amounts of muscle masses, which are MF20 and Mc4r-GFP positive (Fig. 2e-g). This expression pattern of Mc4r-GFP seems correlated to the proceeding of limb muscle development, and continues in later stage limbs (for example, E16.5 and E18.5) (Fig. 2i,j).

**Fig. 3:**
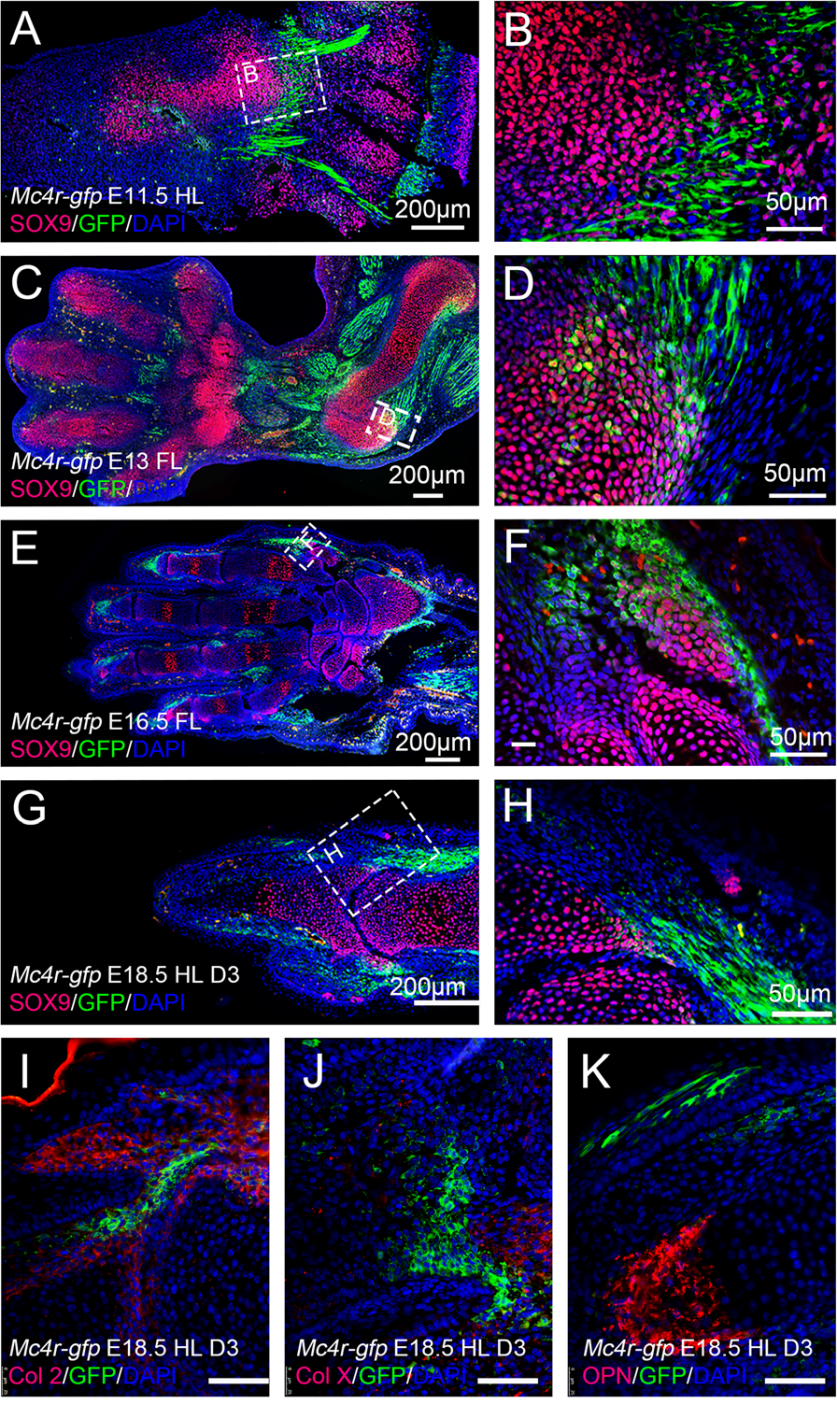
Mc4r-GFP expression in the skeleton of embryonic mouse limb. **a-h** Representative images of Mc4r-GFP and Sox9 expression detected by immunofluorescence staining, on sections of E11.5 hindlimb (**a,b**), E13 forelimb (**c,d**), E16 forelimb (**e,f**), and E18.5 hindlimb digit 3 (**g,h**). (**b,d,f,h**) are higher magnification of outlined areas in (**a,c,e,g**). **i-k** Expression of Mc4r-GFP and Col 2 (**i**), Col X (**j**) and OPN (**k**) on section of E18.5 hindlimb digit 3. Images are shown with distal to the left. Scale bars: 200 μm in (**a,c,e,g)**; 50 μm in (**b,d,f,h, i-k)**

The increased final height and bone mass in individuals with pathogenic *MC4R* mutations suggest that MC4R may have a role in bone turnover (Lepsen et al., 2020; Martinelli et al., 2011). We analyzed whether Mc4r-GFP is expressed in the bone cells. In E11.5 embryos, we detected Sox9 in the dermomyotome in the somites. But most of the Sox9 positive cells are GFP negative (Fig. 3a,b). There is very few Sox9/Mc4r-GFP double positive cells in the E13.5 (Fig. 3c,d), or later stage limbs. In the digits of E16.5 and E18.5 limbs, abundant Mc4r-GFP cells are present, surrounding the skeleton, but only a very small number of cells are also Sox9 positive (Fig. 3e-h). It was reported that *Mc4r* transcript is detectable in human osteoblast-like cell lines (Zhong et al., 2005). But we failed to detect Collagen II (Col 2), Collagen X (Col X), or Osteopontin (OPN) expression in the Mc4r-GFP cells (Fig. i-k). We reasoned that these cells are part of the ligament/tendon, but failed to find suitable antibodies in our analysis.

### Mc4r-GFP expression in the adult mouse digit

**Fig. 4:**
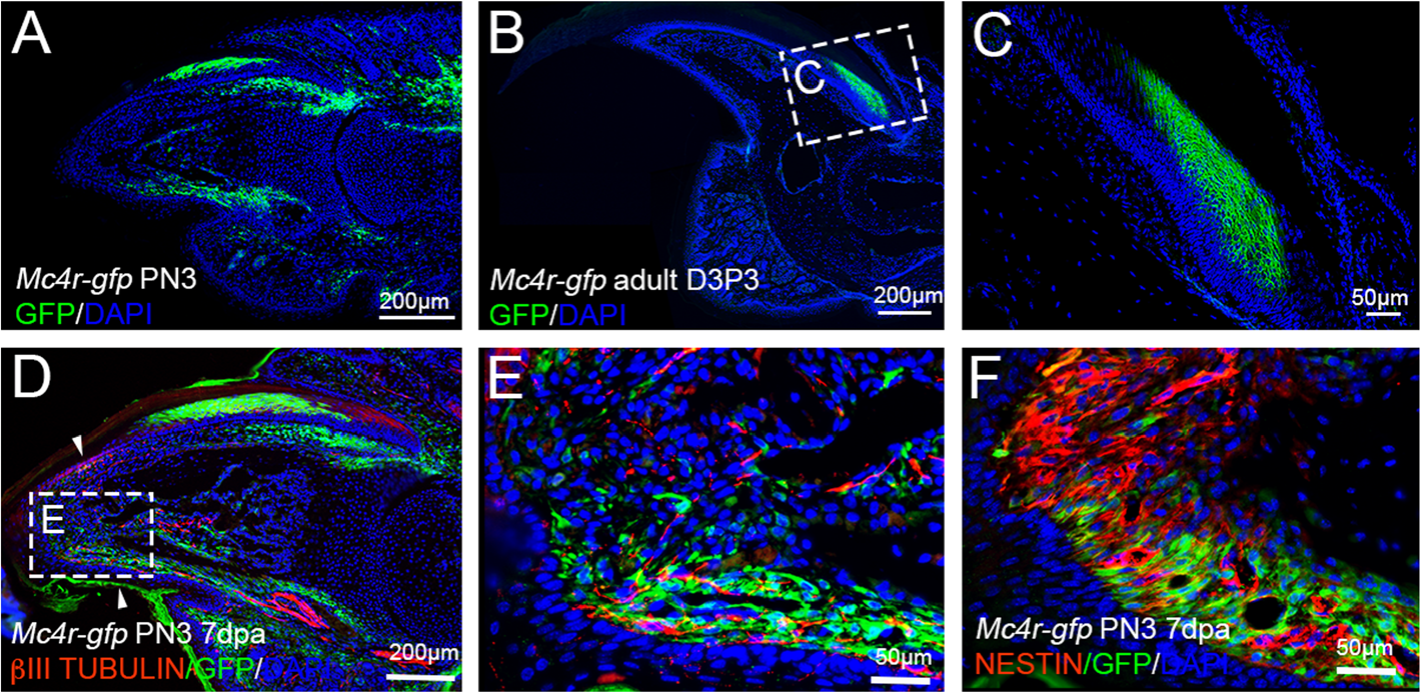
Mc4r-GFP expression in the nail matrix of neonatal and adult digits. **a** Detection of Mc4r-GFP on section of postnatal 3 (PN3) digit 3. **b,c** Mc4r-GFP detection on section of adult digit 3, higher magnification of the nail organ is shown in (**c)**. **d,e** Expression of Mc4r-GFP and βIII-tubulin in the neonatal digit regenerate, 7 days post amputation (dpa). **f** Expression of Mc4r-GFP and Nestin in the neonatal digit regenerate, 7 dpa. Arrowheads in (**d**) indicate amputation level. Scale bars: 200 μm in (**a,b,d),** 50 μm in (**c,e,f)**

Compared to the embryonic limb and digit, Mc4r-GFP expression in non-injured adult digit has more specific patterns. It is highly expressed in the mesenchymal connective tissues surrounding the terminal phalange bone, and in the nail matrix of postnatal 3 (PN3) mice (Fig. 4a). But in the adult digit, Mc4r-GFP expression becomes more specific, restricted to the proximal nail matrix (Fig. 4b,c). This region of Mc4r-GFP expression is similar to the zone of transient-amplifying cells proposed to be important for nail regrowth and digit regeneration (Takeo et al., 2013). Mc4r-GFP expression in the regenerates at 7 days post amputation (dpa) amputated at PN3 has similar pattern observed in the adult digit regenerate (Zhang et al., 2018), with Mc4r-GFP expressing cells in the vicinity of βIII-tubulin and Nestin positive cells (Fig. 4d-f). This expression pattern also supports a close relationship of the MC4R signaling to the function of nerves in regeneration (Zhang et al., 2018).

### Effects of α-MSH on distal digit regeneration in the *Mc4r+/−* mice

**Fig. 5:**
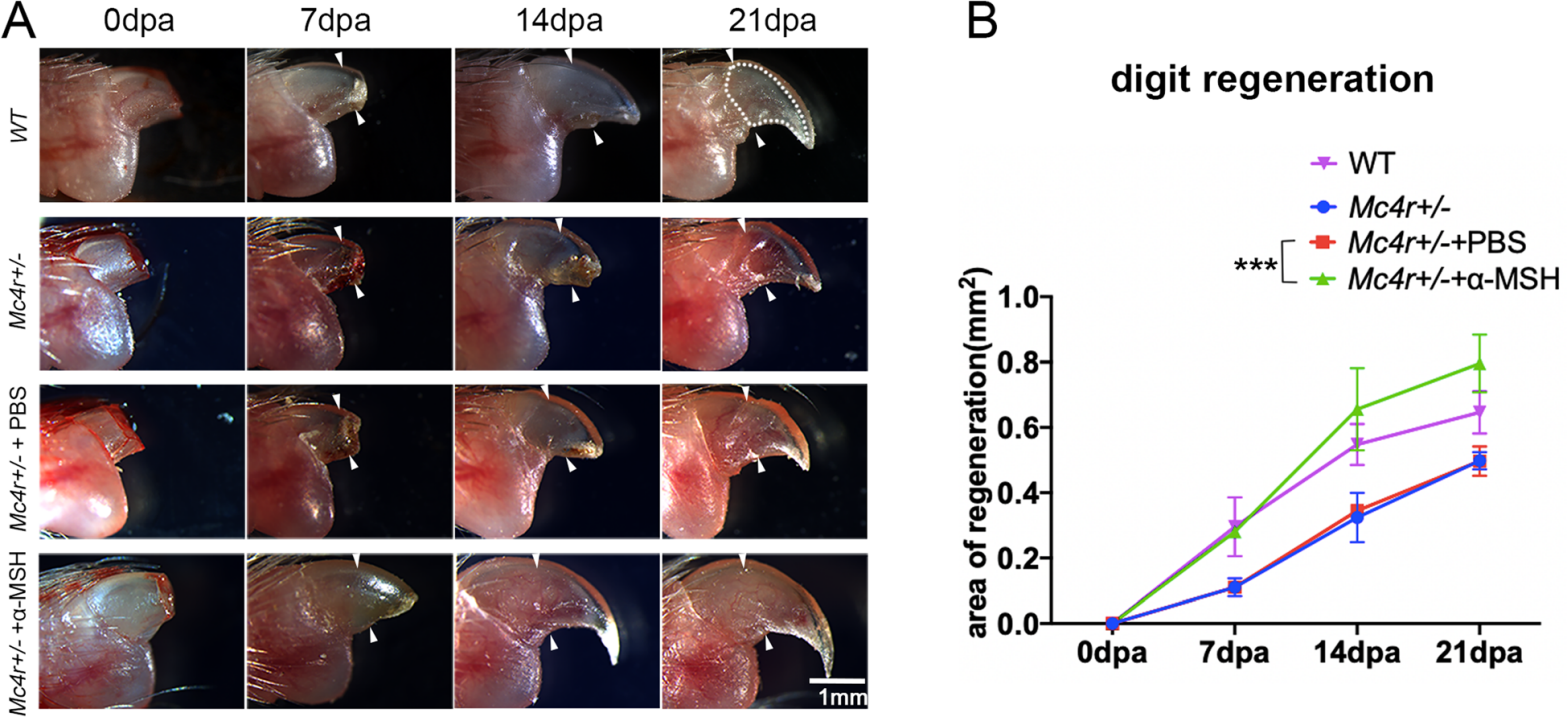
Digit regeneration in *Mc4r+/−* mice rescued by α-MSH injection. **a** Images of wild type (WT) and *Mc4r+/−* mouse digits at 0, 7, 14, 21 days post amputation (dpa), with PBS or α-MSH injection (1 mg/kg, every other day). Arrowheads indicate amputation levels. Scale bar: 1 mm. **b** Measurement of area of digit regeneration. Error bars, standard derivation (*n* = 9 for each group). Students’ *t*-test was used to compare PBS and α-MSH injected groups against control (*Mc4r+/−)*. There was no significant difference of area of regeneration between Mc4r^+/−^ + PBS and Mc4r^+/−^ groups, between WT and Mc4r^+/−^ + α-MSH groups; There was significant difference between Mc4r^+/−^ + PBS and Mc4r^+/−^ + α-MSH groups, between Mc4r^+/−^ + PBS and Mc4r^+/−^ groups (**)

When the terminal phalange of the mouse digit is amputated distally (about 40%), the digit can regenerate (reviewed in Seifert and Muneoka, 2018; Simkin et al., 2013). This distal digit regeneration ability is lost in *Mc4r−/−*, and delayed in *Mc4r+/−* mice (Zhang et al., 2018). To explore whether activating MC4R signaling could rescue digit regeneration, we amputated 40% of the digit tips in *Mc4r+/−* mice and administrated α-MSH by intraperitoneal injection (1 mg/kg, every other day). As shown in Fig. 5, similar to untreated controls, PBS injection had no significant effect on the speed and the scale of digit tip regeneration (Fig. 5a). In contrast, α-MSH injection significantly sped up digit tip regeneration (Fig. 5a). The 7 dpa α-MSH injected digit looked much better than the 14 dpa PBS injected digit. Calculation of the area of regeneration of the digits further confirmed that α-MSH significantly promoted distal digit regeneration in the *Mc4r+/−* mice, to an extent that the regeneration of the α-MSH injected *Mc4r+/−* digit is equivalent to the wild type controls (Fig. 5b).

### Effect of α-MSH on distal digit regeneration after denervation

**Fig. 6:**
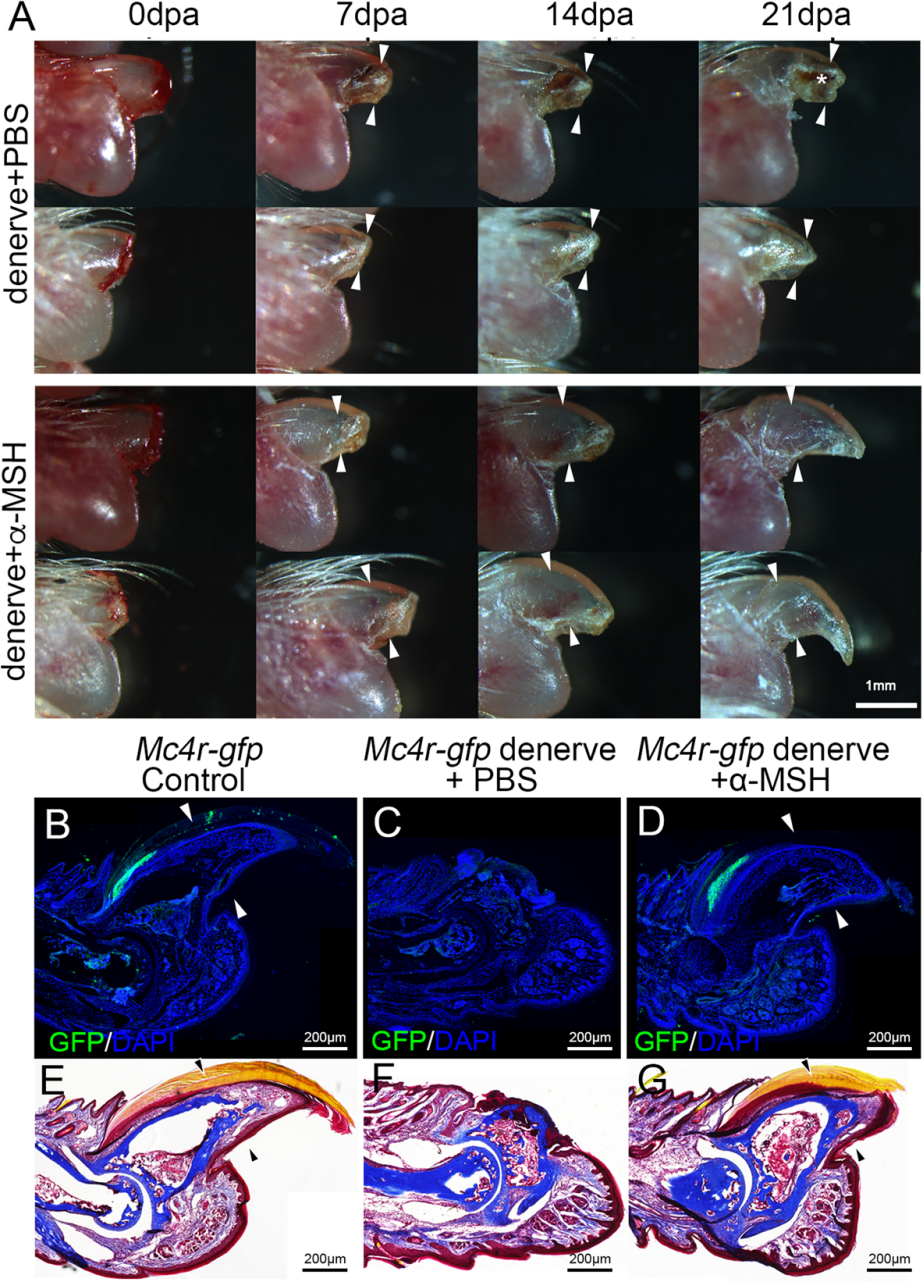
Digit regeneration in denervated hindlimb rescued by α-MSH injection. **a** Images of adult mouse digits at 0, 7, 14, 21 dpa, with PBS or α-MSH injection (1 mg/kg, every other day, *n* = 5 for each group), after removal of the sciatic and femoral nerves. **b-d** GFP expressing nail organ regrown in innervated (**b**), and α-MSH injected, denervated (**d**) digit, but is absent in PBS injected, denervated digit (**c**). **e-g** Trichrome staining on sections of digit stumps from control (**e**), PBS injected, hindlimb denervated (**f**), and α-MSH injected, hindlimb denervated (**g**) digit. Arrowheads in (**a-g**) indicate amputation levels. Scale bars: 1 mm in (**a)**, 200 μm in (**b-g**)

The denervated tadpole limb restores regeneration ability with α-MSH activation of MC4R signaling (Zhang et al., 2018). To examine whether α-MSH also has neurotrophic effect on mouse digit, we denervated the hindlimb of the adult mice, waited for two weeks, and performed distal amputation (40% distal) afterwards, as previously described (Rinkevich et al., 2014). We injected the mice with PBS or α-MSH (1 mg/kg) every other day, and examined regeneration of the digit weekly until 21 dpa. The denervated digits not only failed to regenerate, but also degenerated and receded beyond the amputation level with PBS injection (Fig. 6a, also Fig. 6c,f). The denervated digit stump had persistent scab (white *, Fig. 6a), and no sign of blastema formation (Fig. 6a). But the α-MSH injected, denervated digits could regenerate (Fig. 6a).

As mentioned above, we noticed that the Mc4r-GFP is strongly expressed in the nail matrix in the adult mice. We wondered whether α-MSH have any effect on the nail regrowth, which plays a critical role in digit regeneration (Zhao and Neufeld, 1995). We found that GFP expression is lost in the denervated digit (Fig. 6c), but Mc4r-GFP expression was restored by α-MSH in the denervated digits (Fig. 6d), with a similar pattern to the control (Fig. 6b). Trichrome staining of the digit sections also confirmed the presence of the nail organ in the regenerated digit of α-MSH injected mice (Fig. 6e-g). These results suggested that MC4R signaling have important functions in nail regrowth, which has been shown to be a prerequisite for digit regeneration (Zhao and Neufeld, 1995).

### Effects of α-MSH on proximal digit amputation

**Fig. 7:**
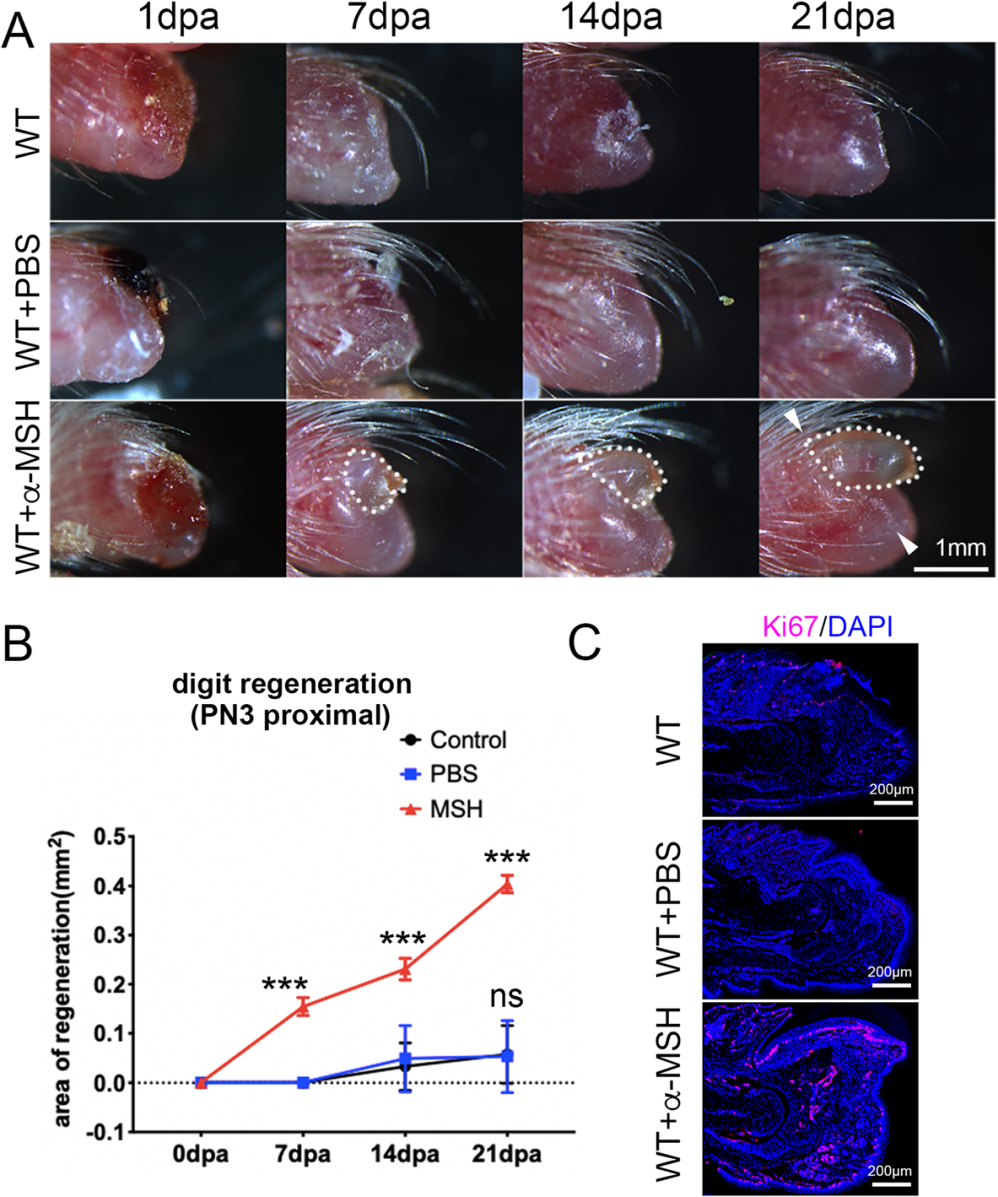
Stimulated proximal digit regeneration in neonatal mice by α-MSH. **a** Images of mouse digits at 0, 7, 14, 21 dpa post proximal digit amputation at PN3, with PBS or α-MSH injection (1 mg/kg, every other day). **b** Analysis of area of digit regeneration shows the induced regeneration in α-MSH treated group, n = 5 for each group. Error bars: standard derivation, Students’ *t*-test was used to compare PBS and α-MSH injected groups, and against control. ns, no significant difference between PBS and Control, *** indicates significant difference, *p* < 0.001, α-MSH injected compared to control group, or PBS group. **c** Ki67 detection on sections of proximally amputated digits, 21 dpa. Proliferating cells (Ki67) are shown in red. Nuclei counterstained with DAPI are shown in blue. Scale bars: 1 mm in (**a**), 200 μm in (**b**, **c**)

We further explored whether activating MC4R signaling could be used to stimulate regeneration under a non-regenerative condition. For this purpose, we performed proximal digit amputations in the PN3 neonates. We found that digit regeneration did occur after α-MSH injection, in contrast to PBS injected and untreated controls (Fig. 7a). Measurement of the area regenerated showed that α-MSH injection significantly induced regeneration (Fig. 7b). Consistently, there were plentiful proliferating cells (Ki67+) accumulated in the regeneration blastema in α-MSH injected mouse digits, but not in PBS injected digits (Fig. 7d). We noted that the stimulated proximal digit regeneration is not as good as distal digit regeneration (for example, compare Fig. 7a to Fig. 5a). Nevertheless, this result supported that α-MSH activation of MC4R could be explored to stimulate proximal digit regeneration.

## Discussion

In this report, we investigated the MC4R signaling in the perspectives of regeneration in one peripheral tissue-the mouse digits. Regeneration of the digit tip has been documented in humans (Douglas, 1972; Illingworth, 1974), and in animal models (Borgens, 1982; Neufeld and Zhao, 1993). This regeneration ability is restricted to the distal part of the terminal phalanges. Understanding the cellular and molecular mechanisms of digit regeneration is important for developing treatment for limb and digit injuries (Seifert and Muneoka, 2018; Storer and Miller, 2020). Based on the expression dynamics of MC4R in the developing limb and digits, and our functional analysis of α-MSH during digit regeneration, we conclude that α-MSH activation of MC4R signaling has a neurotrophic function in digit regeneration.

First, consistent to its high expression in the central nervous system, especially in the ventral spinal cord, mouse Mc4r expression in the embryonic limb and postnatal digit is closely related to the nerves (Fig. 1). As the limb develops, there is an increasing number of Mc4r-GFP expressing cells accumulated in the limb. How Mc4r expression is regulated in the limb is not yet fully investigated, but it appears that most of these non-nerve cells are muscle progenitor cells. And the appearance of Mc4r in the limb muscles corresponds to secondary myogenesis and neuromuscular contact establishment, which occurs around E13.5 in rodents (Hurren et al., 2015). The earlier expression area of Mc4r in somites (E11.5 for example), is also juxtaposed with nerves (Figs. 1, and 2). On the other hand, we did not observe significant expression of Mc4r in the limb skeleton (Fig. 3), although it has been reported that *Mc4r* transcripts are present in bone cell lines (Zhong et al., 2005). This indicates that MC4R does not act locally in bone turnover, consistent to a finding that osteoblast-derived lipocalin 2 (Lcn2) crosses the brain-blood barrier to activate the hypothalamus MC4R neurons (Mosialou et al., 2017).

Second, we show that α-MSH activation of MC4R has stimulating effect on digit regeneration. It has been well demonstrated that in amphibian limb regeneration, proliferation of blastema cells is nerve dependent (reviewed in (Farkas and Monaghan, 2017; Kumar and Brockes, 2012; Stocum, 2019)). In our previous work, we showed that tadpole limb regeneration could be rescued by α-MSH supplementation when the limb is denervated after spinal cord transection. And in the *Mc4r−/−* mice, digit regeneration is completely blocked (Zhang et al., 2018). Both findings suggest a neurotrophic role of MC4R signaling. Here we show that intraperitoneal injection of α-MSH restores distal regeneration of the *Mc4r+/−* mouse digits (Fig. 5), α-MSH administration rescues regeneration of the denervated digits (Fig. 6), and even stimulate regeneration of the proximally amputated digits (Fig. 7). Thus, our results further demonstrate that α-MSH activation of MC4R signaling has neurotrophic function during mouse digit regeneration. This is in agreement with several previous studies. For example, it has been shown that Mc4r expression is upregulated when spinal nerve is injured (van der Kraan et al., 1999), while endogenous α-MSH-like peptides stimulate peripheral nerve regrowth after injury (Plantinga et al., 1995).

It is noteworthy that Mc4r-GFP is distinctively expressed in the nail matrix of the adult mouse digits (Fig. 4). An important observation of digit regeneration in the mouse and rat is that successful digit regeneration correlates with the retainment and regrowth of the nail organ. If the nail is excluded in distal amputation, the digit tip regeneration fails, while proximally amputated digit can regrow the bone if the nail organ is retained (Neufeld and Zhao, 1993; Zhao and Neufeld, 1995). However, the mechanism underlying this correlation remains poorly understood. In 2013, Takeo et al. showed that activation of Wnt signaling in the nail epithelium is required for nail regrowth and for attracting nerves that promote blastema growth and digit regeneration (Takeo et al., 2013). The expression of Mc4r-GFP in the nail matrix correlates to the zone of transient-amplifying cells in the nail matrix (Fig. 4). We note that the distal boarder of Mc4r-GFP expression in the nail matrix seems to mark the 50% level of the terminal phalanges (Fig. 4). We reckon that the difference of the activity of MC4R signaling between the proximal and the distal part of the terminal phalanges, if there is any, deserves further investigation.

Inducing proximal limb regeneration in mammals remains a challenge to overcome. Recently, we have achieved relatively good regeneration in the adult mouse phalange 2 (P2) by transplantation of limb progenitor cells (Chen et al., 2017). It will be worthwhile to combine the various approaches, including activation of MC4R signaling, in the effort for inducing mammalian digit regeneration.

## Conclusion

Mc4r is dynamically expressed during limb development, with significant expression in limb nerves and limb muscles that require neural contacts. Mc4r is required for mouse digit regeneration and its endogenous agonist α-MSH can rescue digit regeneration in the *Mc4r+/−* mice. α-MSH activation of MC4R signaling has neurotrophic function in mouse digit regeneration, stimulating digit regeneration in denervated hindlimb, and inducing proximal digit regeneration. Thus, the potential of using α-MSH activation of MC4R signaling for inducing digit regeneration merits further investigation.

## Methods

### Animal care and husbandry

Animal care and husbandry was in accordance to guidance of the Association for Assessment and Accreditation of Laboratory Animal Care International (http://www.aaalac.org/index.cfm). All animal procedures were approved by the Institutional Animal Care and Use Committees (IACUC) at the Tongji University and the University of Minnesota. The *Mc4r-gfp* (*B6.Cg-Tg(Mc4r-MAPT/Sapphire)21Rck/J*) transgenic mice strain was originally generated by Dr. Jeffrey Friedman’s lab (Liu et al., 2003) and is available at Jax laboratory (RRID:IMSR_JAX:008323). The *B6;129S4-Mc4r*^*tm1Lowl*^*/J* (*Mc4r+/−*) mouse strain, originally generated by Lowell lab (Balthasar et al., 2005), was obtained from Jax laboratory (RRID:IMSR_JAX:006414).

### Mouse digit amputation, denervation of mouse Hindlimb, and α-MSH injection

For GFP expression and immunofluorescence analysis during digit regeneration. The digit 3 phalange 3 (D3P3) was amputated at distal (40% distally) or proximal (more than 50% from the distal tip) level under anesthesia. Both male and female mice, at age of postnatal 3 (PN3) or 12-week-old (adult), were used. For assessment of α-MSH administration and denervation, *Mc4r+/−*, *Mc4r-gfp* and wild type mice, both male and female, were used.

Denervation of the mouse lower hindlimb was achieved by sciatic and femoral nerve transection, as previously described (Rinkevich et al., 2014). Briefly, mice were anesthetized with Avertin. The dorsal and ventral hairs from the posterior thigh were shaved and the skin was prepared with povidone iodine solution and 75% alcohol. An incision of the skin parallel with the femur was made with sharp scissors, and the muscles were bluntly divided, parallel to and just inferior to the femur, to expose the sciatic nerve. About 5 mm of the nerve was removed to prevent healing and regeneration of the nerve terminus. The incision was closed with 6–0 absorbable sutures. Femoral nerve was transected similarly. Success of hindlimb denervation was determined by significant retarded movement of the operated hind limb. A 2-week period between denervation and digit amputation minimized the opportunity of nerve supply to the digit stumps.

Following proximal digit amputation, or distal amputation in the denervated mice, α-MSH (1 mg/kg) was injected intraperitoneally, every other day, for 2 weeks. Body weight and area of digit regeneration were measured at 0, 7, 14, 21dpa (days post amputation).

### Immunofluorescence and Trichrome staining

Embryonic *Mc4r-gfp* mice were used for GFP expression and immunofluorescence analysis was carried out as described (Zhang et al., 2018). Briefly, samples were fixed in Leica 4% paraformaldehyde (PFA) (Sigma), and decalcified in 0.4 M EDTA in PBS for several days (except embryonic stages). Samples were embedded in optimum cutting temperature (OCT) compound (for immunofluorescence), or paraffin (for trichrome staining), and sectioned.

For immunofluorescence, sections were permeabilized with 1% Triton X-100 in PBS for 15 min, and blocked with blocking buffer (PBS containing 1% BSA and 0.3% Triton X-100). Primary antibodies were incubated overnight at 4 °C, and secondary antibodies were incubated for 90 min at room temperature. Slides were counterstained with 4,6-diamidino-2-phenylindole (DAPI), and mounted. Primary antibodies used were MF20 (ebioscience,4,301,341), GFP (Abcam, ab290), Sox9 (Abcam, ab185966), β-III-tubulin (Sigma T2200), α-MSH (Sigma-Aldrich, AB5087), Nestin (R&D Systems, MAB2376), Ki67 (Abcam, ab16667). Secondary antibodies were Alexa Fluor 488, Alexa Fluor 555, Alexa Fluor 647-conjugated IgGs (Invitrogen, Carlsbad, CA, USA).

For Masson trichrome staining, sections were de-paraffined and pretreated in Bouin’s fluid at 56 °C for 1 h before they were stained with Trichrome staining kit (Sigma HT15-1KT) according to the manufacture’s instruction. The slides were mounted with Permount mounting medium (Fisher).

### Quantitation of regeneration and statistical analysis

Images of mouse digits were taken at 0, 7, 14, 21dpa with a digital camera (Leica DFC 450) attached to a Leica DM16FC microscope. The areas of regenerated part (PN3 proximal amputation) were selected and the pixels were counted with the histogram function of Photoshop (Adobe). The pixels numbers were then converted to areas (for example, for an image taken with 5 × magnification, with size of 2560 × 1920 pixels, the calibration is 1 mm = 919 pix). For the digits after denervation, the area distal to the nail folds at 0dpa was subtracted from 7, 14, 21 dpa (Fig. 7b). GraphPad Prism (https://www.graphpad.com/) was used to analyze all data. Results were presented at Mean with Standard Derivation (error bars). Unpaired *t*-tests were used for comparison of means of data between groups, and one-way ANOVA was used for analysis of the whole groups. Differences were considered significant if *p*-value < 0.05(*), < 0.01(**), or < 0.001(***).

## Supplementary Information

**Additional file 1: Fig. s1** Analysis of body weight changes after digit amputation, compared to WT group. Error bars, standard derivation, *n* = 9. Students *t*-test was used to compare groups against control (WT). * indicates significant difference, *p* < 0.05, ** indicates significant difference, *p* < 0.01. ns: no significant difference.
