# Supplementary figures and images for: Activation of the Melanocortin-4 receptor signaling by α-MSH stimulates nerve-dependent mouse digit regeneration
Source: Cell Regen. 2021 May 3;10:19. doi: 10.1186/s13619-021-00081-9 (PMC8089069; doi:10.1186/s13619-021-00081-9)

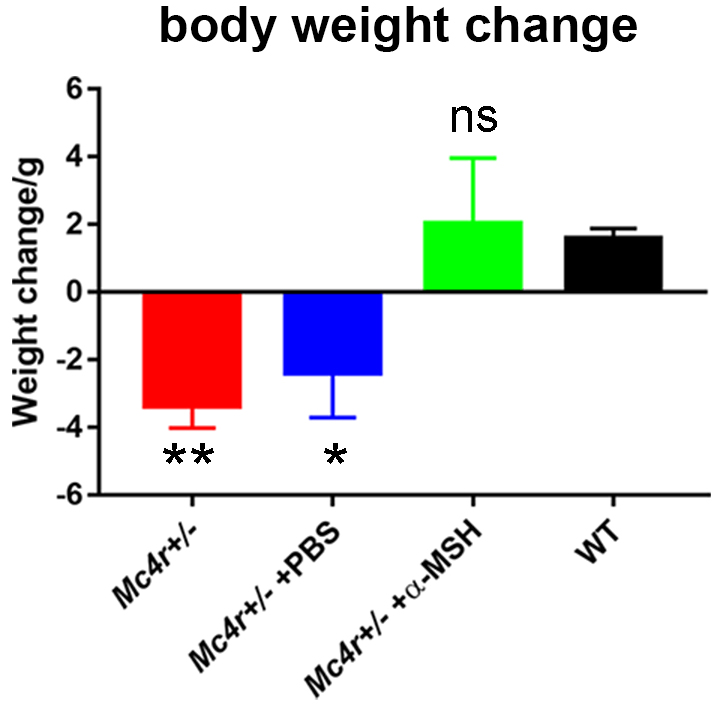

Supplement: Supplementary file 1 — Additional file 1: Fig. s1 Analysis of body weight changes after digit amputation, compared to WT group. Error bars, standard derivation, n = 9. Students t-test was used to compare groups against control (WT). * indicates significant difference, p < 0.05, ** indicates significant difference, p < 0.01. ns: no significant difference. [file 13619_2021_81_MOESM1_ESM.jpg]
